# Description of Virulent Factors and Horizontal Gene Transfers of Keratitis-Associated Amoeba Acanthamoeba Triangularis by Genome Analysis

**DOI:** 10.3390/pathogens9030217

**Published:** 2020-03-16

**Authors:** Issam Hasni, Julien Andréani, Philippe Colson, Bernard La Scola

**Affiliations:** 1 Institut de Recherche pour le Développement IRD 198, Aix-Marseille Université UM63, Assistance Publique – Hôpitaux de Marseille (AP-HM), Microbes, Evolution, Phylogeny and Infection (MEΦI), Institut Hospitalo-Universitaire (IHU) - Méditerranée Infection, 13005 Marseille, France; issemhasni@gmail.com (I.H.); miaguiabidou@gmail.com (J.A.); philippe.COLSON@univ-amu.fr (P.C.); 2 R&D Department, Amoéba, 38 Avenue des Frères Montgolfier, 69680 Chassieu, France

**Keywords:** free-living amoebae, *Acanthamoeba triangularis*, genome, genes, keratitis, pathogenicity

## Abstract

*Acanthamoeba triangularis* strain SH 621 is a free-living amoeba belonging to *Acanthamoeba* ribo-genotype T4. This ubiquitous protist is among the free-living amoebas responsible for *Acanthamoeba* keratitis, a severe infection of human cornea. Genome sequencing and genomic comparison were carried out to explore the biological functions and to better understand the virulence mechanism related to the pathogenicity of *Acanthamoeba* keratitis. The genome assembly harbored a length of 66.43 Mb encompassing 13,849 scaffolds. The analysis of predicted proteins reported the presence of 37,062 ORFs. A complete annotation revealed 33,168 and 16,605 genes that matched with NCBI non-redundant protein sequence (nr) and Cluster of Orthologous Group of proteins (COG) databases, respectively. The Kyoto Encyclopedia of Genes and Genomes Pathway (KEGG) annotation reported a great number of genes related to carbohydrate, amino acid and lipid metabolic pathways. The pangenome performed with 8 available amoeba genomes belonging to genus *Acanthamoeba* revealed a core genome containing 843 clusters of orthologous genes with a ratio core genome/pangenome of less than 0.02. We detected 48 genes related to virulent factors of *Acanthamoeba* keratitis. Best hit analyses in nr database identified 99 homologous genes shared with amoeba-resisting microorganisms. This study allows the deciphering the genome of a free-living amoeba with medical interest and provides genomic data to better understand virulence-related *Acanthamoeba* keratitis.

## 1. Introduction

*Acanthamoeba* species are ubiquitous free-living amoebas (FLA) widely distributed in diverse environments, including fresh water, ocean sediment, dust, soil, and air [1]. These protists are causative agents of *Acanthamoeba* keratitis (AK), a serious infection of the cornea associated with trauma, exposure to contaminated water, and contact lens use [2,3,4,5]. This infection is frequently encountered around the world and causes vision loss in case of misdiagnosis, inadequate use of lens-contact carrier, or inappropriate treatment [3,4,5]. The pathogenicity mechanisms of AK have been reported and divided into direct and indirect factors [2]. The direct factors characterize the ability to adhere to epithelial cells of the host cornea by the expression of acanthopodia and adhesin proteins [6,7]. Once the host tissue is penetrated, amoebas phagocyte the epithelial cornea and secrete toxins, leading to inducible cell apoptosis [6]. The indirect factors are related to different characteristics, including the ability to encyst, morphology of the protist, or drug resistance [2]. Based on the 18S rRNA gene, the genus *Acanthamoeba* is defined and distributed into 22 different ribo-genotypes (T1–T22) [5,8]. Most of the human infections due to Acanthamoeba are related to ribo-genotype T4 [9]. Indeed, several studies have estimated that 90% of AK amoebic keratitis cases have been linked to this genotype [9]. *Acanthamoeba triangularis* strain SH 621 is a ubiquitous FLA belonging to genotype T4, the most pathogenic group of *Acanthamoeba* species [10]. *A. triangularis* has a two-stage life, switching between a trophozoite form and a cyst form. The *A. triangularis* trophozoite is an active stage in which the amoeba moves, feeds, and divides. Under harsh unfavorable conditions, such as changes in temperature, pH, lack of nutrients, treatment with disinfectant or therapeutic agents, the amoebas have the ability to adopt a resistant double-walled cyst stage [11]. Free-living amoeba (FLA) food microorganisms, by selective grazing to grow, contribute to the regulation of the environmental microbial population [1,12]. Despite that, some microorganisms named amoeba-resisting microorganisms (ARMs), such as bacteria, fungi, or giant viruses, have the capacity to bypass the internalization in order to survive and multiply in amoebas [13,14,15,16]. The close contact between FLA and ARM promotes horizontal gene transfers, leading to the evolution of microorganisms [17,18].

In this study, we explore the *A. triangularis* genome sequenced with NextSeq Illumina technology. The gene content was analyzed by a comparison of the set of protein sequences with the main public databases. Then, we performed pan-genomic and core genomic analyses within the *Acanthamoeba* species. Through comparative genomic analysis, we gain insight into the potential pathogenicity pathways in the setting of *Acanthamoeba* keratitis. Finally, we study horizontal gene transfers with ARMs.

## 2. Results

### 2.1. General Genomic Features

The trimmed reads resulted in 8,540,421 reads used for the assembly of the *A. triangularis* genome. The draft genome size of *A. triangularis* obtained represents 66,434,030 nucleotides (nt). The genome was assembled into 13,849 scaffolds. For comparison with other *Acanthamoeba* available in public databases, the genome length of *A. triangularis* is larger than the genome of *A. castellanii* Neff (42.02 Mb) and *Acanthamoeba polyphaga* Linc-AP1(49.35Mb), that was assembled in 2013 [19] and 2019 [20], respectively. However, the *A. triangularis* genome size is smaller than the genome size of *Acanthamoeba castellanii* ATCC 50370 (120.6Mb) and *Acanthamoeba polyphaga* ATCC 30872 (115.3Mb), assembled in 2015 [21]. 

The genome harbored a guanine–cytosine (GC) content of 58.6%, which is similar to the GC content of *A. polyphaga* (58.1%) and *A. castellanii* (57.8%) [20]. The main *A. triangularis* characteristics are presented in Table 1.

We detected one complete 18S rRNA gene of *A. triangularis* with a length of 1748 nucleotides. Moreover, several other partial sequences related to 18S rRNA of *A. triangularis* were found in the genome in different or similar scaffolds (Figure S1).

Phylogenetic analysis based on the 18S rRNA gene showed that *A. triangularis* is most closely related to the *Acanthamoeba* spp. belonging to genotype T4, including *Acanthamoeba castellanii* strain CDC:0786:V042 (U07403.1), *Acanthamoeba polyphaga* strain HC-2 (AF019056.1), and *Acanthamoeba* genotype T4 isolate Aud6 (KF733230.1) (Figure S2). A total of 37,062 putative genes were predicted (Table 2). 

We found a larger number of predicted proteins for *A. triangularis* than for *A. castellanii* Neff (20,681 ORFs) [19]. In contrast, we found a smaller number of genes predicted for *A. triangularis* than the genome of *A. castellanii* ATCC 50370 (82,310 ORF) [21], which is explained by the difference in genome length. 

### 2.2. Functional Annotation

In order to conduct an in-depth analysis of the genetic content of *A. triangularis,* the putative proteins were compared with different databases (Table S1). 

**Figure 1:**
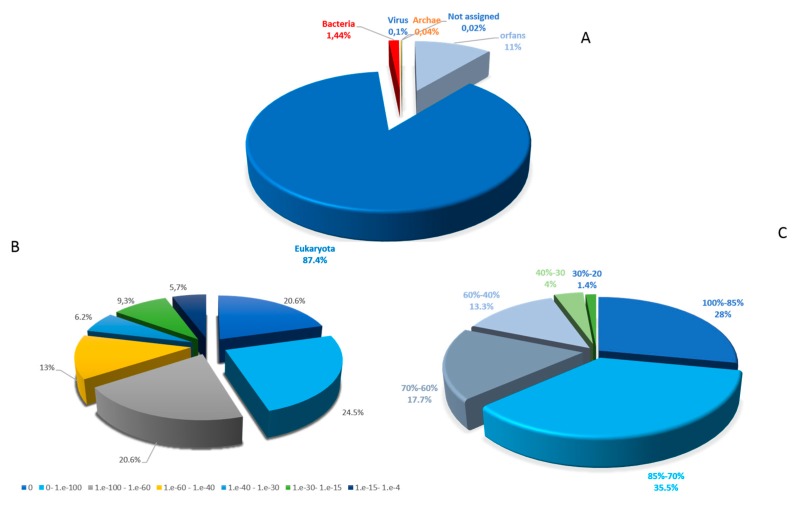
Characteristics of similarity search of each *Acanthamoeba triangularis* protein sequences assigned to a function in NCBI non-redundant protein sequence (nr) database. (**A**) taxonomical distribution. (**B**) E-value distribution. (**C**) Similarity distribution.

The BLASTp analysis revealed that 33,168 (89%) protein sequences have a hit in the nr database and 4094 (11%) are classified as ORFans. The taxonomical distribution of the sequences matched against the nr database revealed that a large proportion of the predicted protein sequences were shared with eukaryotes (32,567: 98.2%), followed by bacteria (537: 1.6%), viruses (35: 0.11%), archaea (15: 0.05%) and unassigned organisms (14: 0.04%) (Figure 1A). Among the best BLASTp hits with eukaryotes, 30,548 sequences (83.5%) are shared with *A. castellanii* strain Neff (ATCC 30010), an amoeba that can cause human keratitis and which is phylogenetically close to *A. triangularis*. The e-value distribution analysis of the genes in the nr database revealed that 65.8% of sequences had a very high homology, as indicated by an E-value <10^− 60^, whereas only 5.7% of the sequence had a best BLAST with an E-value ranging from 1e^-15^ and 1e^-4^ (Figure 1B). The similarity distribution of the hits in the nr dataset indicated that 81% of the sequences had a similarity higher than 60%, while only 19% of sequences had a similarity less than 5.5% (Figure 1C). The mean length of protein sequences was at 374 amino acids.

**Figure 2:**
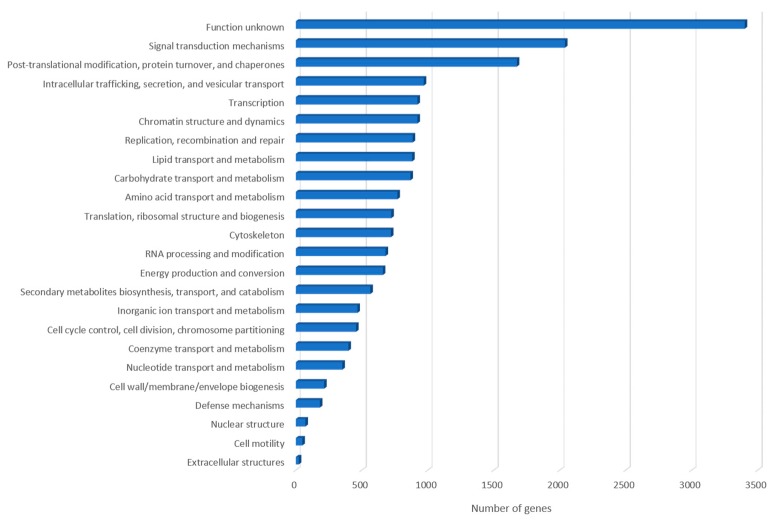
Representation of *A. triangularis* genes related to different clusters of gene categories.

To obtain more information on gene functions, we searched with BLAST and the predicted proteins against the Cluster of Orthologous database (COG). The COG investigation had assigned 16,605 genes (44.8%) to the COG classification and distributed in 23 COG groups. The cluster for “unknown function” (3402: 20.5%) was the largest represented followed by “signal transduction mechanisms” (2040: 12.3%), “post-translational modification, protein turnover and chaperones” (1675: 10.1%), “intracellular trafficking secretion, and vesicular transport” (970: 5.8%) and “transcription” (922: 5.6%). Among the COG functional classes, the categories “cell motility” (50: 0.3%) and “extracellular” (23: 0.1%) were less represented (Figure 2). 

**Figure 3:**
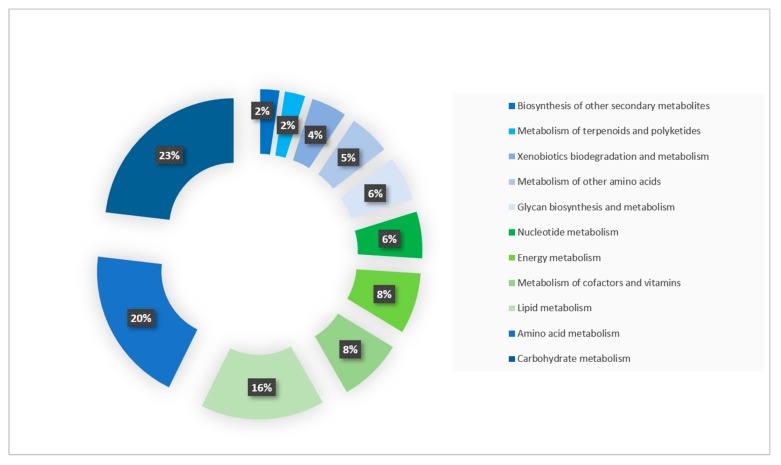
Distribution of *A. triangularis* protein sequences involved in the metabolic pathway. The protein of *A. triangularis* was compared to the Kyoto Encyclopedia of Genes and Genomes Pathway (KEGG) database and the repartition of these protein sequences in the metabolic pathway was visualized.

To better understand the biological functions and metabolic pathways of protein sequences, we performed an enrichment analysis with KEGG pathway database. Among the 37,062 protein sequences of *A. triangularis*, 10,101 (27%) were assigned to a function in KEGG database and were matched in 348 KEGG pathways. A large part of proteins (3228: 32%) were involved in metabolic pathways and were classified into 11 subcategories. The genes involved in carbohydrate metabolism (747) were the most abundant entries followed by genes implicated in amino acid metabolism (632) and lipid metabolism (502). Furthermore, we found 1628, 1864, and 1943 proteins sequences mapped with genetic information processing, environmental information processing, and cellular process, respectively (Figure 3).

### 2.3. Gene Related to Keratitis Pathogenicity

**Figure 4:**
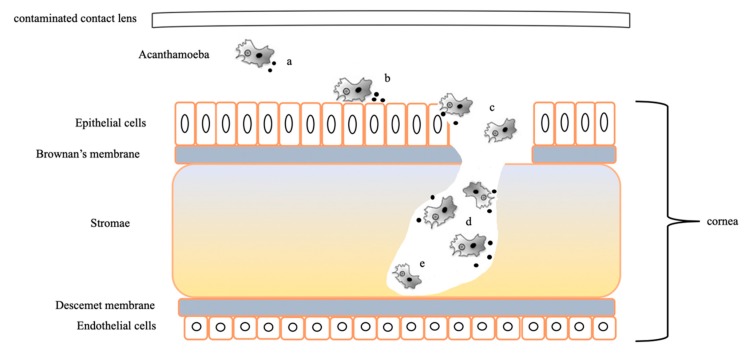
Infection of human cornea by *Acanthamoeba* species. (a) *Acanthamoeba* detached from contaminated lens. (b) *Acanthamoeba* spp. attached and adhered to epithelial cells by receptor (mannose receptors and glycoproteins) and secreted metalloproteases to digest external environment. (c) *Acanthamoeba* spp. destroyed epithelium and Brownan’s membrane in order to penetrate within stroma environment (secretion of proteases, glycosidases, and hydrolytic enzymes). (d) Destruction of stromae. (e) Radial keratoneuritis (an infiltrate along the corneal nerves).

The identification of genes possibly related to keratitis virulence was performed by a genomic comparative study between *Acanthamoeba* spp. and different amoebas not involved in keratitis pathogenesis. The analysis reported 314 clusters containing 1004 orthologs within the genus *Acanthamoeba*. Among these 314 clusters, 48 genes are related to the pathology of *Acanthamoeba* keratitis (Table S2). The virulent factors causing keratitis are divided into two classes, including the factors contributing directly and indirectly to *Acanthamoeba* pathogenicity [6] (Table S2 and Table 3; Figure 4).

Among the primary factors, we found 45 genes related to virulence. We detected the presence of mannose-inducted protein (MIP), a gene encoding an important transmembrane protein involved in the adhesion to the surface of the cornea [27]. We found 17 genes related to cytoskeleton, especially 3 genes encoding for actin-binding protein. The analysis reported the presence of 8 lipases, especially 3 phospholipases playing a potential role in membrane disruption and host cell lysis [6]. The survey showed the presence of various genes encoding peptidases (n = 11), which are enzymes that facilitate host invasion [6]. Furthermore, we reported the presence of 1 glycosidase. The study exhibited the presence of a peroxidase and a glutathione peroxidase that are antioxidant enzymes involved in the amoeba defense against reactive oxygen species. Among the factors indirectly related to pathogenicity, we found numerous heat shock protein genes (3 genes) involved in high-temperature survival [6] (Table 3). All these genes potentially related to the pathogenesis of keratitis were not clustered in a common region of the *A. triangularis* genome.

### 2.4. Investigation on Sequences Inherited from Potential Horizontal Gene Gransfers

The analysis of the best BLASTp hits for *A. triangularis* predicted proteins revealed the presence of 99 hits in amoeba-resistant microorganisms (ARMs), including amoeba-resistant bacteria (ARBs), fungi, amoeba endosymbionts, and giant viruses (Table S3). Among the 99 ARM best matches with *A. triangularis*, we identified 62 genes shared with bacteria that could multiply or survive within amoebae. The majority of ARB sequences belonged to the obligate intracellular bacteria of the *Chlamydiae* phylum. Indeed, we reported 44 best hits (involving 44.4% of ARM genes shared with *A. triangularis*) belonging to *Chlamydia* members that lysis *Acanthamoeba* species or live in endosymbiosis within amoebas. We found sixteen genes (16.2%) best matching with bacteria isolated in amoebae from environmental samples or able to survive in-vitro within amoebae such as *Acinetobacter* spp. and *Pseudomonas* spp. The analysis revealed the presence of two hypothetical sequences with best hits belonging to *L. pneumophila*, a pathogenic human bacterium leading to respiratory illness. Finally, two sequences (cleavage stimulation factor subunit 2 and mitochondrial import inner membrane translocase subunit TIM9) of *A. triangularis* are shared with *Cryptococcus neoformans*, a human pathogenic fungi in immunocompromised patients that can invade and multiply within *A. castellanii* [28].

Of the 99 ARM homologs identified in the genome of *A. triangularis*, 37 have best matched with viruses, including 35 with giant viruses (35.4%). Most of these viral sequences are shared with *Pandoraviridae* members. Indeed, we identified 19 genes belonging to 5 different *Pandoravirus* strains (*P. quercus*, *P. inopinatum*, *P. macleodensis*, *P. neocaledonia,* and *P. salinus*) with *A. triangularis* best match. These 19 genes encode 9 hypothetical proteins, 1 F-box domain-containing protein, 1 methyltransferase, 1 ribonuclease BN, 2 ribonucleoside-diphosphate reductase small chain, 1 signal peptidase I, 1 metallophosphatase, 1 morn repeat protein, 1 transp Tc5 C and 1 serine/threonine protein kinase. Furthermore, we identified 5 *A. triangularis* homologous sequences shared with Medusavirus, a giant virus that replicates on *Acanthamoeba castellanii*, which was isolated from hot spring water [29]. Five of the thirty-five genes with best match to the giant viruses belonged to members of the *Mimiviridae*, including Mimivirus, Tupanvirus, and Catovirus. The identification of one *A. triangularis* gene best matching with Marseillevirus sequence encoding hypothetical protein was reported (Table S3). Other homologs of *A. triangularis*, shared with giant viruses, belonged to Pithovirus sibericum and Mollivirus sibericum, which were isolated from Siberian permafrost [30,31]. A further functional enrichment by COG revealed that most of the *A. triangularis* genes shared with ARM organisms were assigned an unknown function (11 genes), followed by amino acid transport and metabolism (4 genes), carbohydrate and metabolism (3 genes), and coenzyme transport and metabolism (3 genes) (Figure S3). 

The study of putative horizontal transfers was evaluated by phylogenetic reconstructions based on the *A. triangularis* homologs with ARMs. Among the 99 potential genes shared with ARMs, we were able to obtain 82 phylogenetic trees. The protein sequences with insufficient numbers of hits did not allow us to examine the potential horizontal transfers, as this was the case for 17 genes. The phylogenetic analyses showed that lateral gene transfer were confirmed for 62 (62.6%) protein sequences, including 34 and 28, which had a best hit with organisms belonging to ARBs and giant viruses, respectively.

**Figure 5:**
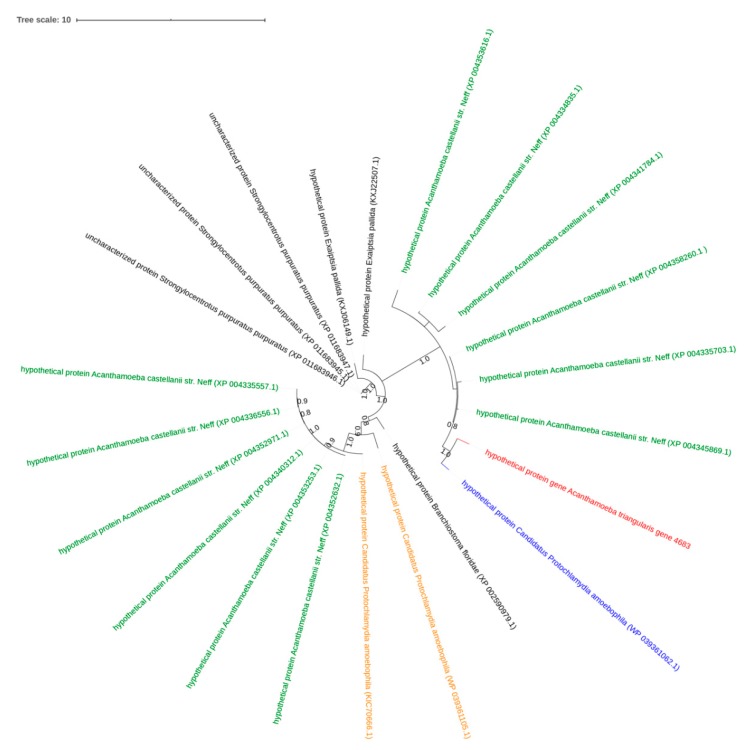
Representation of horizontal transfer analysis. Phylogenetic tree for *A. triangularis* protein of putative ARM origin. The tree was constructed using maximum-likelihood method based on hypothetical protein sequences of *A. triangularis*. The tree was performed with 21 homologous sequences of *A. triangularis* retrieved by BLASTp on NCBI. In red: hypothetical protein of *A. triangularis* SH621; in blue: the closest homolog from *Candidatus Protochlamydia amoebophila*; in orange: other homologs from chlamydia; in green: homologs from other amoebas; in black: homologs from other organisms.

**Figure 6:**
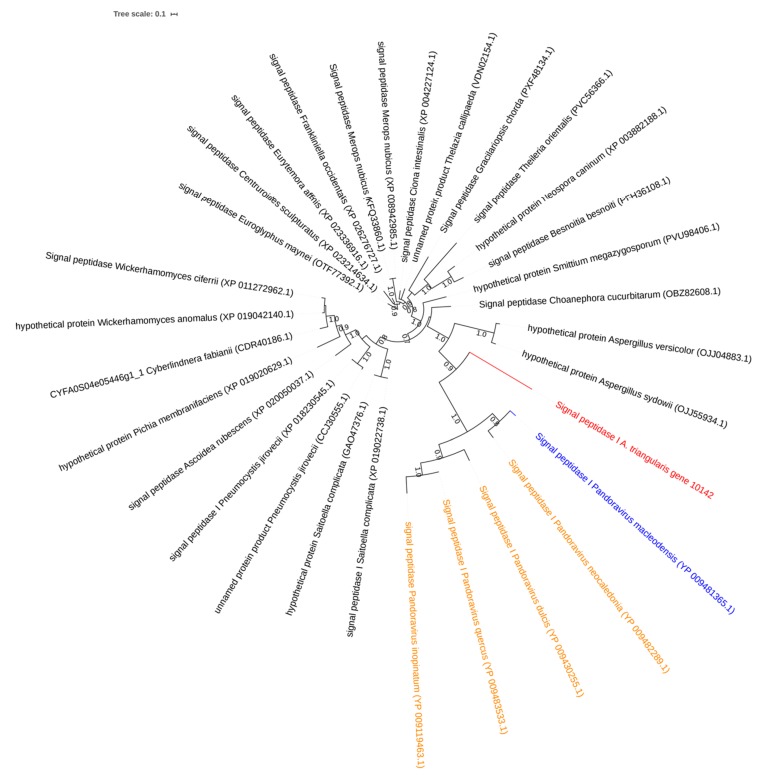
Representation of horizontal transfer analysis. Phylogenetic tree for *A. triangularis* protein of putative ARM origin. The tree was constructed using maximum-likelihood method based on the signal peptidase I sequences of *A. triangularis*. The tree was performed with 30 homologous sequences of *A. triangularis* retrieved by BLASTp on NCBI. In red: signal peptidase I of *A. triangularis*; in orange: homologs from ARM (Pandoravirus strains); in black: homologs from other organisms.

A hypothetical protein of *A. triangularis* (gene 4683) shared homologs with *Candidatus Protochlamydia amoebophila* and *A. castellanii,* an amoeba phylogenetically related to *A. triangularis*. Furthermore, the phylogeny tree based on this hypothetical protein of *A. triangularis* and these ARM homologs showed that the closest homolog to the *A. triangularis* gene identified so far was found in *Candidatus Protochlamydia* and suggests that the gene was transferred from *A. triangularis* to *Candidatus Protochlamydia* (Figure 5). The BLASTp analysis revealed that the signal peptidase I gene of *A. triangularis* (gene 10142) was homologous with 4 Pandoravirus strains. Moreover, the phylogeny tree showed clustering of signal; these peptidase I homologs suggest a putative horizontal gene transfer from Pandoraviruses to *A. triangularis* (Figure 6).

### 2.5. Pan-Genome and Core Genome Analyses of Acanthamoeba spp.

**Figure 7:**
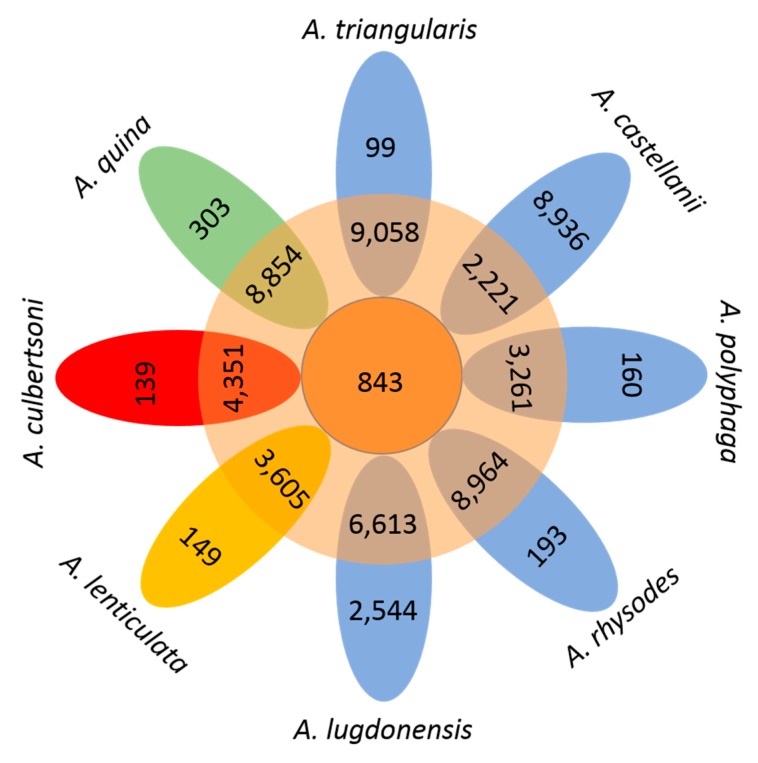
Flower plot showing the core, dispensable, and strain-specific genes of the 8 *Acanthamoeba* species. The flower plot displays the core gene number (in the center), the dispensable gene number (in the annulus), and the strain-specific gene number (in the petals) for the 8 *Acanthamoeba* species. The numbers under the strain name denote the total number of related genes. Different colors indicate different *Acanthamoeba* T-genotype groups: T4 genotype in blue; T5 strains in yellow; T10 strains in red; T11 strains in green.

The genome of 8 different *Acanthamoeba* species was compared in order to analyze the genetic diversity between the different *Acanthamoeba* species. *Acanthamoeba* pangenome size reaches 59,450 genes encompassing clusters or unique genes (Figure 7). 

A total of 843 clusters composed the core genome, which represented 1.5% of the pangenome. Among the core genome, *A. triangularis* exhibited 399 clusters (0.7%) with two representative sequences and 227 clusters (0.4%) composed of three representative sequences (Table S4). Moreover, *A. triangularis* genes were part of 99 clusters of unique genes (Table S4). The COG analysis showed that the “unknown function”, “post-translational modification, protein turnover, and chaperones” function, and “signal transduction mechanisms” function (63) are among the categories the most represented for core genome and *A. triangularis* unique sequences (Figure S4 and Figure S5). Surprisingly, we found a large number of unique genes for *Acanthamoeba castellanii* (n = 8,236 genes) and *Acanthamoeba lugdunensis* (n = 2544). These unique genes play a role in signal transduction, metabolism pathway, and DNA biosynthesis (Figure S6). However, a large part of these genes is categorized as unknown function (233 and 137 for *A. castellanii* and *A. lugdunensis*, respectively). This high number of unique genes could be explained by the larger predicted protein number of *A. castellanii* (73,447) and *A. lugdonensis* (65,171) compared to the other amoebae used in the analysis (*Acanthamoeba culberstoni*, 22,241; *Acanthamoeba lenticulate*, 29,468; *Acanthamoeba polyphaga*, 32,524; *Acanthamoeba quina*, 49,881; *Acanthamoeba rhysodes*, 47,088 and *A. triangularis*, 39,411). Finally, a phylogenetic tree based on the presence and absence of homologous genes within the *Acanthamoeba* pangenome showed that *A. triangularis* was clustered with *A. rhysodes* (Figure S7).

## 3. Discussion

Members of the genus *Acanthamoeba* are amoebae in which we have a particular interest because they are involved in human infections and have a potential role as vectors for pathogenic microorganisms [3,5,13]. Currently, the characterizing of *Acanthamoeba* spp. is based on ribosomal sequences and only two *Acanthamoeba* genomes have been deep investigated, especially *A. castellanii* and *A. polyphaga* [19,20,32,33]. 

In our study, we explored the genomic content of *A. triangularis* strain SH 621, an amoeba belonging to *Acanthamoeba* ribo-genotype T4, related to human keratitis. The genomic approach provided the main characteristics of this protist with a significant genome of more than 66.43 Mb encompassing 13,849 scaffolds. *A. triangularis* harbored a genome size approximatively 2-fold smaller than *A. castellanii* ATCC 50370 (120.6 M) and *A. polyphaga* ATCC 30872 (115.3 Mb). The size of *A. castellanii* ATCC 50370 and *A. polyphaga* ATCC 30872 is composed of large number of contigs (*A. polyphaga*; 224,482 scaffolds, A. *castellanii* 221,748 scaffolds) with very short sequences, suggesting a potential overestimation of the size genome and an assembly mistake.

Moreover, *A. castellanii* Neff (42.02 Mb) and *A. polyphaga* Linc-AP1(49.35Mb) [20] have a genome size of 1.7- and 1.3-fold smaller than the *A. triangularis*, respectively. These differences in sizes can be explained by a sequencing technology and assembly tools used, which were different between the sequenced *Acanthamoeba* genome. Currently, the characterizing of *Acanthamoeba* is based on ribosomal sequences. However, we observed that *Acanthamoeba* organism can contain multiple copies of 18S rRNA genes, including one complete and 3 incompletes. This aspect could be a source of heterogeneity and represents a potential limit of ribo-typing. So, genomic analysis could provide more information in order to classify the *Acanthamoeba* species.

The comparison of 37,062 predicted protein sequences against the three main public databases allowed us to obtain a detailed annotation and better knowledge on the biologic function of *A. triangularis* genes. The annotation revealed a composition from diverse putative origins of the draft genome sequences. These results show a great proportion with eukaryotic organisms, especially with *A. castellanii* strain Neff ATCC 30010. The analysis based on *A. triangularis* virulence reported 48 genes related to keratitis mechanisms. We found gene encoding for mannose-binding proteins (MBP), important proteins that mediate the amoeba adhesion to the corneal epithelial cells [34,35]. The adhesion of amoebas to the host cells is an essential step in the pathogenesis of *Acanthamoeba* keratitis. Several studies reported the central pathogenic property of the MPB and its involvement in the destruction of target cells [27,34,35,36]. MBP had been potential targets to develop therapeutic antibodies in order to inhibit the host–parasites interaction [37]. We also identified various proteases directly related to the pathogenicity of *Acanthamoeba* spp., especially serine protease and metalloproteases that contribute to the evasion of the host. The analysis revealed the presence of 3 phospholipases shared with potentially pathogenic *Acanthamoeba* species. The role of phospholipase in the membrane disruption, entrance in the host cells, and cell lysis was suspected in several studies [6,38]. Furthermore, phospholipase involvement has been reported in the induction of inflammatory responses facilitating the *Acanthamoeba* virulence [6]. The genomic study showed the presence of heat shock protein (Hsps) specific to pathogenic *Acanthamoeba* species. Hsps are essential to survive and adapt organisms at higher temperatures in order to maintain their metabolic activities within the host [39,40]. So, the genomic analysis is a useful strategy to identify and characterize potential targets to develop a new therapeutical approach.

BLASTp and phylogenetic analyses revealed horizontal gene transfers between *A. triangularis* and some pathogenic bacteria, such as *L. pneumophila*, a human respiratory pathogen that multiplies and lysis *Acanthamoeba* spp. [41]. Among the ARM organisms, it was the “*Chlamydia* endosymbionts” organisms that had the highest number of homologs with *A. triangularis*, including *Protochlamydia amoebophila* and *Neochlamydia* organisms. These homolog genes could be exchanged during horizontal endosymbiont transmission. Out of 99 putative genes exchanged with ARMs, 49 genes were virus-infecting amoebas. The proportion of giant viral sequences within *A. triangularis* (0.13%) was smaller compared to *A. castellani* Neff (1.2%) and *W. magna* c2c maky (0.3%) [33,42]. However, the proportion was higher compared to the giant virus sequences contained in the genome of *A. polyphaga* (0.1%) [21]. The presence of viral sequences within *A. triangularis* genome is consistent with the history of giant viruses which is related to the genus *Acanthamoeba.* Indeed, since the isolation of the first giant virus named “*Acanthamoeba polyphaga mimivirus*” from *A. polyphaga*, the *Acanthamoebas* species have been used as support to isolate giant viruses by co-culture [43,44,45,46,47]. These amoebae seemed to be more permissive compared to other families of amoebae since a large part of the giant viruses have been isolated from *A. castellanii* and *A. polyphaga*, including Mimivirus, Marseillevirus, Pandoravirus, and Tupanvirus [43,45,46,48]. The majority of viral sequence genes best matching with *A. triangularis* were viruses invading amoebas from the *Acanthamoeba* genus. Furthermore, we observed that the large part of exchanged genes is assigned a function, which could bring a benefit to the organism that receives the sequences.

These sequence exchanges have shown that *Acanthamoeba* organisms represent a biological niche that contributes to the genome heterogeneity of FLA. Moreover, the low part of the core genome (0.02) compared to the pangenome demonstrated the plasticity of *Acanthamoeba* genomes.

Therefore, the study provides better knowledge on the FLA of Amoebozoa clade and insight into the unexplored world of FLA. The proximity between FLA and microorganisms favor horizontal gene transfers. In addition, pathogenicity analysis has revealed that some virulence genes related to keratitis are specific to the genus *Acanthamoeba*. 

## 4. Materials and Methods

### 4.1. Culture of Acanthamoeba Triangularis Strain SH 621

The culture of *A. triangularis* (ATCC 50254^TM^) was performed at 30 °C using 175 cm² culture flasks in PYG medium (Thermo Fisher Scientific, Illkirch, France) [49]. When the trophozoites formed a monolayer, the amoebas were detached from the flask and harvested by centrifugation at 700× *g* for 10 min, followed by three washing steps using Page’s modified Neff’s Amoeba Saline medium (2 mM NaCl, 16 μM MgSO_4_, 27.2 μM CaCl_2_, 1 mM Na_2_HPO_4_, 1 mM KH_2_PO_4_). Amoeba quantification was performed using a KOVA^®^ slide cell counting chamber.

### 4.2. Extraction and Sequencing of DNA

DNA of *A. triangularis* was extracted with 1 volume of phenol/chloroform/isoamyl alcohol (50:49:1) (Merck KGaA). The mix was centrifugated 5 min at 10,000× *g* and we recovered the aqueous phase. We repeated twice this DNA extraction step. Then, we precipitated the DNA with the addition of 2 volumes of ethanol (Merck KGaA) and gently stirred by hand. The suspension was incubated overnight at −20 °C, centrifuged at 10,000× *g* for 30 min at 4 °C, the supernatant was removed and the DNA solution dried at room temperature. Finally, DNA was dissolved in 0.5 mL in TE buffer (10 mM Tris, 0.1 mM EDTA). Genomic DNA (gDNA) of *Acanthamoeba triangularis* was quantified by a Qubit assay with the high sensitivity kit (Life Technologies, Carlsbad, CA, USA) to 7.1 ng/µL. Genomic DNA was next sequenced on the MiSeq Technology (Illumina Inc, San Diego, CA, USA) with the paired-end strategy. To prepare the paired-end library, a dilution was performed to require 1 ng of genome. The tagmentation step fragmented and tagged the DNA. Then a limited number of cycle PCR cycles (12) completed the tag adapters. After purification on AMPure XP beads (Beckman Coulter Inc, Fullerton, CA, USA), the libraries were then normalized on specific beads according to the Nextera XT protocol (Illumina). Normalized libraries were pooled into a single library for sequencing on the MiSeq. The pooled single strand library was loaded onto the reagent cartridge and then onto the instrument along with the flow cell. Automated cluster generation and paired-end sequencing with dual index reads were performed in a single 39-h run in 2 × 250-bp. Total information of 3.4 Gb was obtained with a cluster density of 360,000 per mm^2^ and, finally, with a cluster passing quality control filters of 96.8%. Within this run, the index representation for *A. triangularis* was determined at 63.84%. The 4,302,070 paired-end reads were filtered according to the read qualities. 

### 4.3. Genome Assembly

The quality of raw data from DNA sequencing was controlled using FastQC software (https://www.bioinformatics.babraham.ac.uk/projects/fastqc/). Raw data were trimmed with the Trimmomatic software [50]. Indeed, the reads with low quality were removed and only the reads with average quality above 28 were selected. All trimmed reads of DNA were assembled de novo using CLC Genomics Workbench v7.51 (https://www.qiagenbioinformatics.com/products/clc-genomics-workbench/). The 64-word size and 100 bubble size parameters were used. The contigs with size under 980 bp were removed. To identify and remove scaffolds that were likely to have originated from bacterial or viral contaminants, we conducted BLASTn searches against the local databases with the megablast option [51]. The assembly was improved with GapFiller [52]. The quality assessment of the genome assembly was analyzed using QUAST software [53]. The genome was deposited in Genbank under the number accession CACVKS010000000. Then, a phylogenetic analysis based on the 18S rRNA gene was performed. The 18S rRNA gene of *A. triangularis* strain SH 621 was identified by BLASTn comparison between the amoebal genome assembly and the 18S rRNA sequences of *A. triangularis* (AF316547.2) available in the NCBI GenBank nucleotide sequence database (nt). The 18S rRNA gene of *A. triangularis* strain SH 621 was deposited in Genbank (LR757994). Homologs were searched for using BLASTn against the nt database. Multiple sequence alignment was carried out using MUSCLE software [54]. Finally, a phylogenetic analysis of these nucleotide sequences was performed using MEGA version 7 and the maximum likelihood (ML) algorithm, with 1000 bootstrap replicates [55].

### 4.4. Functional Annotation

The open reading frame prediction was performed using AUGUSTUS, a software optimized for the eukaryotic genome [56]. The biological function of *A. triangularis* predicted proteins was analyzed by a comparison of their sequences with those from public databases, including the NCBI non-redundant protein sequence database (nr), Cluster of Orthologous Group of proteins (COG) database and against Kyoto Encyclopedia of Genes and Genomes Pathway (KEGG) database. Firstly, the function of protein sequences was identified by a BLASTp search against the nr database with E-value cutoff at 1e-03 [51]. The COG annotation was performed using EggNOG [57,58] with diamond as mapping mode. To analyze the metabolic pathway and the biological function of the genes, we have mapped the *A. triangularis* sequences against the Kyoto Encyclopedia of Genes and Genomes Pathway [59].

### 4.5. Analysis of Virulence Related Genes 

To identify genes possibly related to *Acanthamoeba* keratitis, we performed comparative genomic analysis with *Acanthamoeba* species (*Acanthamoeba culberstoni*: CDFF01000001.1, *Acanthamoeba lenticulata*: NAVB01000001.1, *Acanthamoeba polyphaga:* LQHA01000001.1, *Acanthamoeba lugdunensis*: CDFB01000001.1, *Acanthamoeba quina*: CDFN01000001.1, *Acanthamoeba rhysodes*: CDFC01000001.1, *Acanthamoeba castellanii*: CDFL01000001.1, and *Acanthamoeba triangularis*: CACVKS010000000) and other amoebas, including non-pathogenic amoebas (*Willaertia magna*: PRJEB30797, *Naegleria gruberi*: GCA_000004985.1, *N. lovaniensis*: GCA_003324165.1, *Dictyostelium discoideum*: PRJNA13925, and *Acytostelium subglobosum*: PRJNA280978) and pathogenic amoebas (*Entamoeba histolitica*: PRJDB4673 and *Naegleria fowleri*: GCA_000499105.1) that are not involved in keratitis. All *Acanthamoeba* spp. selected for the analysis belong to the species and ribo-genotypes related to keratitis in humans [2,10,60,61,62,63]. In order to identify only the genes related to virulence among these belonging to *Acanthamoeba* spp., we included other amoebas that are not related to keratitis pathogenesis. The genes possibly related to the pathogenicity of keratitis were identified according to a single criterion; they are shared only among the opportunistic amoebae of the genus *Acanthamoeba* used in the study that have been described as being associated with keratitis in the literature [2,3,6,10]. First, we recovered protein sequences of *Dictyostelium discoideum* (PRJNA13925), *Acytostelium subglobosum* (PRJNA280978), and *Entamoeba histolitica* (PRJDB4673) in the Genbank database. For the other amoebas, we performed a protein prediction using the AUGUSTUS program [56]. Then, Proteinortho v5 software was used with these 15 amoeba genomes using as parameters, 60% coverage and 50% amino acid identity, and an e-value of 1e-4 as significance thresholds. To identify the genes related to keratitis mechanisms, we recovered only the homologous genes belonging to 8 *Acanthamoeba* species (*A. culberstoni*, *A. lenticulata*, *A. polyphaga*, *A. lugdunensis*, *A. quina*, *A. rhysodes*, *A. castellanii,* and *A. triangularis*) that were not considered as orthologous genes with others FLA protein sequences used in the analysis. Then, we analyzed the function of these genes shared between *Acanthamoeba* spp.

### 4.6. Study of Horizontal Gene Transfers Between A. triangularis and ARMs

We identified the *A. triangularis* predicted proteins best matching with proteins from amoeba-resistant microorganisms. For each of these protein sequences, we performed a BLASTp search against the NCBI non-redundant (nr) protein sequence database, with an e-value cutoff 1e-03. To confirm the potential horizontal transfers for the genes revealing a best match with amoeba-resistant microorganism homologs, we carried out for a phylogenetic reconstruction all these genes. The alignment of protein sequences were performed using MUSCLE [54]. Phylogenetic trees were obtained using FastTree software [64] and by maximum likelihood method with Jones–Taylor–Thornton (JTT) model with MEGA 7.0.25 software. Phylogenetic trees were visualized using iTOL v3 online [65].

### 4.7. Comparative Genomic Analyses

To identify the best reciprocal hits between different amoebas genomes of the genus *Acanthamoeba*, we used Proteinortho v5 with 60% coverage and 50% amino acid identity, and an e-value of 1e-4 as significance threshold [66]. The clustering was obtained from the predicted proteins using the AUGUSTUS program for eight *Acanthamoeba* species (*Acanthamoeba culberstoni*: CDFF01000001.1, *Acanthamoeba lenticulata*: NAVB01000001.1, *Acanthamoeba polyphaga:* LQHA01000001.1, *Acanthamoeba lugdunensis*: CDFB01000001.1, *Acanthamoeba quina*: CDFN01000001.1, *Acanthamoeba rhysodes*: CDFC01000001.1, *Acanthamoeba castellanii*: CDFL01000001.1 and *Acanthamoeba triangularis*: CACVKS010000000). The functional enrichment was carried out by comparison of protein sequences against COG databases. Finally, pangenome tree based on genus *Acanthamoeba* was generated using the GET_HOMOLOGUES package with the standard parameters [67]. The phylogenetic tree was visualized using iTOL v3 online [65].

## Figures and Tables

**Table 1:** Summary of the *A. triangularis* genome.

Parameter	Number
Haploid genome size (bp)	66,434,030
Sequence contigs (n)	13,849
GC-content (%)	58.6
Maximal scaffold size (bp)	80,033
Minimal scaffold size (bp)	980
Average scaffold size	5322
N50	8852
N75	4376

bp, base pairs; N50, 50% of the genome assembly is, as contigs, larger than this size; N75, 75% of the genome assembly is, as contigs, larger than this size.”

**Table 2:** Comparison of the main genomic features of several amoebas.

Organisms	Genome Size (Mb)	Predicted Proteins	Annotated Proteins	G+C %
*Acanthamoeba triangularis* ATCC 50254	66	37,062	33,168	58.6
*Acanthamoeba castellanii* ATCC 50370	42	20,681	15,455	57.8
*Willaertia magna* C2c Maky	37	18,519	13,571	25
*Naegleria fowleri* ATCC 30863	30	17,252	16,021	35
*Naegleria gruberi* NEG-M	41	15,727	9090	33
*Naegleria lovaniensis* ATCC 30569	31	15,195	13,005	37
*Dictyostelium discoideum* AX4	34	13,541	8422	22
*Entamoeba histolytica* strain HM-1: IMSS	21	8201	4076	24

Source of data: Acanthamoeba castellanii ATCC 50370 [19], Naegleria fowleri [22], Naegleria gruberi [23], Naegleria lovaniensis [24], Dictyostelium discoideum [25], and Entamoeba histolytica [26].

**Table 3:** List of genes possibly related to the pathogenesis in *Acanthamoeba* keratitis.

Gene Identification	Function
	**Adhesion**
gene 34934	mannose binding
	**Metalloproteases**
gene 12288	Aminopeptidase I zinc metalloprotease (M18)
gene 1047	metalloenzyme superfamily
gene 9969	metallocarboxypeptidase
gene 3258	metalloenzyme
	**Proteases**
gene 19397	peptidase S8 and S53 subtilisin kexin sedolisin
gene 3757	PFAM peptidase T2 asparaginase 2
gene 8789	peptidase C19 family
gene 9969	metallocarboxypeptidase
gene 11390	peptidase S8 and S53, subtilisin, kexin, sedolisin
gene 12288	Aminopeptidase I zinc metalloprotease (M18)
gene 12995	peptidase M17 family
gene 25924	Serine aminopeptidase, S33
gene 26649	peptidase C19 family
gene 27916	peptidase C19 family
gene 9026	peptidase C19 family protein
	**Temperature tolerance**
gene 6737	Hsp20/alpha crystallin family
gene 1586	Hsp70 protein
gene 8645	Hsp20/alpha crystallin family protein
	**Phospholipases**
gene 8337	phospholipase A2 activator activity
gene 27752	phospholipase D
gene 34693	phospholipase D
	**Antioxidant defense**
gene 15075	glutathione peroxidase
gene 31764	peroxidase
gene 27487	oxidoreductase

## Supplementary Materials

The following are available online at https://www.mdpi.com/2076-0817/9/3/217/s1. Table S1: Functional annotation analysis of Acanthamoeba triangularis protein sequences; Table S2: List of Acanthamoeba triangularis protein sequences possibly related to keratitis pathogenicity; Table S3: List of Acanthamoeba triangularis protein sequences shared with amoeba-resisting microorganisms; Table S4: List of specific genes and genes of core genome of Acanthamoeba triangularis. Figure S1: Identification of 18S rRNA gene of A. triangularis by comparison against nt database on NCBI. Figure S2: Phylogenetic analysis of amoebas of A. triangularis strain SH 621. Figure S3: Representation of COG functional categories of the A. triangularis sequences shared with ARMs. Figure S4: Representation of COG functional categories of the core genome. Figure S5: Representation of COG functional categories of the unique genes of A. triangularis. Figure S6: Representation of COG functional categories of the unique genes of Acanthamoeba castellanii and Acanthamoeba lugdunensis. Figure S7: Representation of pangenomic tree based on genus Acanthamoeba.
