# Supplementary material for: Description of Virulent Factors and Horizontal Gene Transfers of Keratitis-Associated Amoeba Acanthamoeba Triangularis by Genome Analysis
Source: Pathogens. 2020 Mar 16;9(3):217. doi: 10.3390/pathogens9030217 (PMC7157575; doi:10.3390/pathogens9030217)
Supplement: Supplementary file 1 [file pathogens-09-00217-s001.zip › Supplementary data.docx]

**Title:** Description of virulent factors and horizontal gene transfers of *Acanthamoeba triangularis* by analysis its genome.

**Authors:** Issam Hasni^1,2^, Julien Andréani^1^, Philippe Colson ^1^ and Bernard La Scola^1^*

**Supplementary table legends:**

**Table S1:** Functional annotation analysis of *Acanthamoeba triangularis* protein sequences.

**Table S2:** List of *Acanthamoeba triangularis* protein sequences possibly related to keratitis pathogenicity.

**Table S3:** List of *Acanthamoeba triangularis* protein sequences shared with amoeba-resisting microorganisms.

**Table S4: :** List of specific genes and genes of core genome of *Acanthamoeba triangularis*.

**Figure S1**: Identification of 18S rRNA gene of *A. triangularis* by comparison against nt database on NCBI.


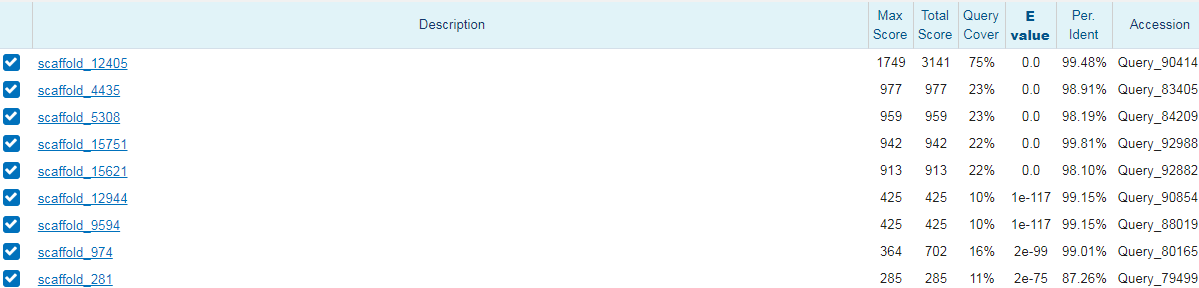

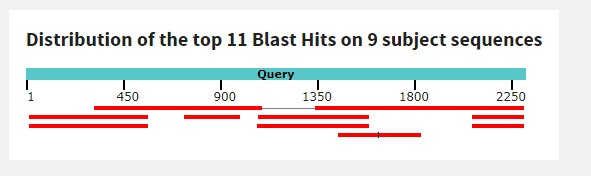


**Figure S2**: Phylogenetic analysis of amoebas of *A. triangularis* strain SH 621. The phylogenetic tree is based on amoeba partial available SSU rRNA sequences of amoebas. GenBank Accession numbers are indicated in parentheses. The sequences were aligned by Muscle and tree was performed using Jukes-Cantor model on MEGA 7.0.25 software. Numbers at the nodes are percentages of bootstrap values obtained by repeating the analysis 1,000 times to generate a consensus tree; only values with bootstraps ≥ 0,7 were displayed.

*Acanthamoeba lugdunensis* (AF005995.1)

*Acanthamoeba* sp. KA/E26 (EF140627.1)

*Acanthamoeba* sp. KA/E12 (AF316545.1)

*Acanthamoeba polyphaga* HC-2 (AF019056.1)

*Acanthamoeba castellanii* CDC : 0786 : V042 (U07403.1)

Acanthamoeba genotype T4 isolate Aud6 (KF733230.1)

*Acanthamoeba* genotype T4 isolate S6 (KF733251.1)

*Acanthamoeba* genotype T4 isolate ASao (KF733223.1)

*Acanthamoeba* genotype T4 isolate Sri1 (KF733257.1)

*Acanthamoeba* genotype T4 isolate S11 (KF733253.1)

*Acanthamoeba* genotype T4 isolate AV (KF733224.1)

*Acanthamoeba* genotype T4 isolate Pat9 (KF733243.1)

*Acanthamoeba triangularis* strain SH 621

*Acanthamoeba* sp. Jin-E5 (AF019054.1)

*Acanthamoeba* sp. isolate U/E8R (AY026251.1)

*Acanthamoeba* sp. 1 FRC-2013 (KF318460.1)

*Acanthamoeba* sp. KA/MSG4 (AY173004.1)

*Acanthamoeba rhysodes* BCM: 0685:116 ATCC 50368 (U07406.1)

*Acanthamoeba* genotype T4 voucher OSU10-025 (JX423611.2)

*Acanthamoeba* sp. KA/MSG26 (AY173010.1)

*Acanthamoeba palestinensis* (AF260719.1)

*Acanthamoeba* sp. ATCC 50496 (U07408.1)

*Acanthamoeba* sp. KA/E28 (EF140629.1)

*Acanthamoeba* sp. strain E6d (GU808294.1)

*Acanthamoeba culbertsoni* Diamond (AF019057.1)

*Acanthamoeba* sp. KA/MSS8-1 (AY173000.1)

*Acanthamoeba castellanii* strain 4CL (AF260724.1)

*Acanthamoeba* sp. UWE2 (AF352388.1)

*Acanthamoeba* sp. strain SCL-14-12 (MK124588.1)

*Acanthamoeba castellanii* ATCC 50374 (U07413.1)

*Acanthamoeba castellanii* strain ATCC 30011 (KF318462.1)

*Acanthamoeba* sp. KA/E22 (EF140638.1)

*Acanthamoeba* sp. KA/E8 (AY148956.1)

*Acanthamoeba* sp. CANZ (JQ271667.1)

*Acanthamoeba* sp. isolate U/E10 (AY026749.1)

*Acanthamoeba royreba* Oak Ridge ATCC 30884 (U07417.1)

*Acanthamoeba divionensis* strain AA2 (AY351646.1)

*Vermamoeba vermiformis* strain MG1 (KU519742.1)

77

99

0.020

**Figure S3**: Representation of COG functional categories of the *A. triangularis* sequences shared with ARMs.

**Figure S4**: Representation of COG functional categories of the core genome.

**Figure S5**: Representation of COG functional categories of the unique genes of *A. triangularis*.

**Figure S6**: Representation of COG functional categories of the unique genes of *Acanthamoeba castellanii and Acanthamoeba lugdunensis*.

**
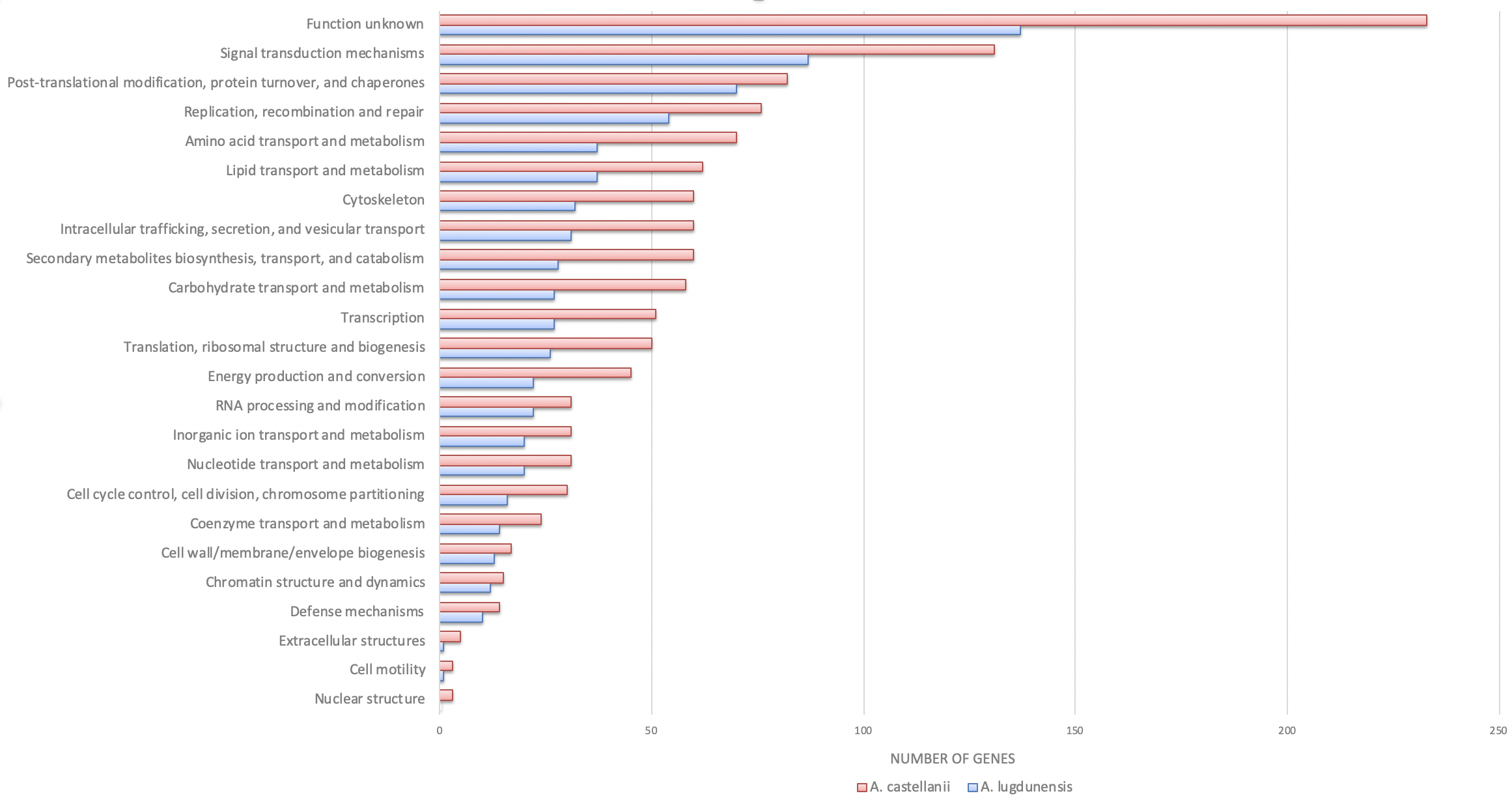
**

**Figure S7**: Representation of pangenomic tree based on *genus Acanthamoeba*.

**
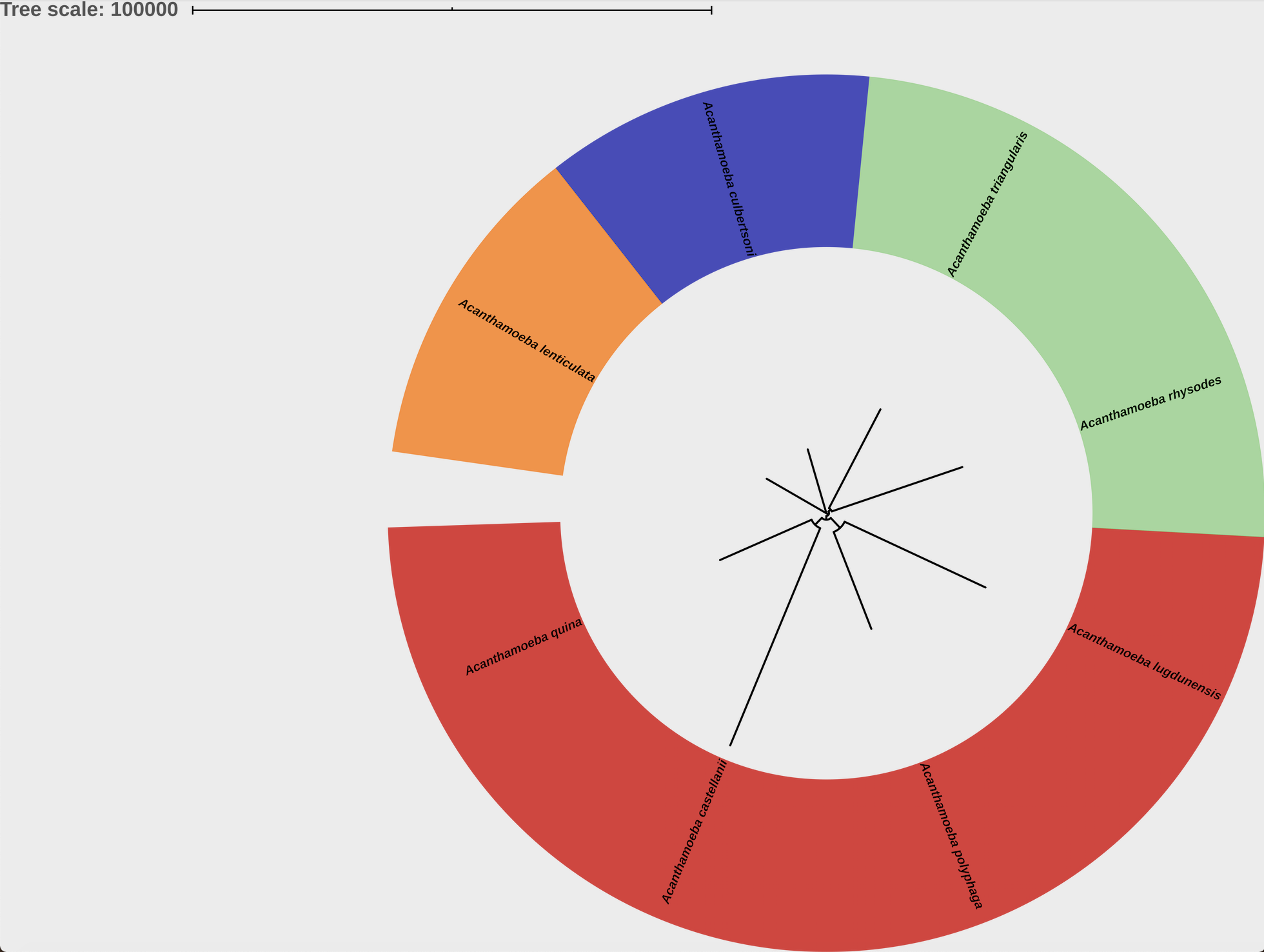
**
